# The evolution and adaptation of A-to-I RNA editing

**DOI:** 10.1371/journal.pgen.1007064

**Published:** 2017-11-28

**Authors:** Arielle L. Yablonovitch, Patricia Deng, Dionna Jacobson, Jin Billy Li

**Affiliations:** 1 Stanford University, Department of Genetics, Stanford, California, United States of America; 2 Stanford University, Biophysics Program, Stanford, California, United States of America; University of Michigan, UNITED STATES

## Abstract

Adenosine-to-inosine (A-to-I) RNA editing is an important post-transcriptional modification that affects the information encoded from DNA to RNA to protein. RNA editing can generate a multitude of transcript isoforms and can potentially be used to optimize protein function in response to varying conditions. In light of this and the fact that millions of editing sites have been identified in many different species, it is interesting to examine the extent to which these sites have evolved to be functionally important. In this review, we discuss results pertaining to the evolution of RNA editing, specifically in humans, cephalopods, and *Drosophila*. We focus on how comparative genomics approaches have aided in the identification of sites that are likely to be advantageous. The use of RNA editing as a mechanism to adapt to varying environmental conditions will also be reviewed.

## Introduction

Transcriptional modifications contribute to transcriptome diversity and flexibility, without the need to make hard-wired mutations at the DNA level. One important transcriptional modification is adenosine-to-inosine (A-to-I) RNA editing, performed by adenosine deaminases acting on RNA (ADAR), a family of enzymes that bind to double-stranded RNA (dsRNA) and deaminate adenosine to form inosine [1]. Inosine is translated as guanosine, so A-to-I editing can alter the amino acid sequences of proteins, as well as affect other transcriptional processes like alternative splicing and microRNA (miRNA) binding [2–4].

RNA editing is a very dynamic process, with editing levels varying from 0% to 100%, which can potentially be affected by environmental stimuli [5,6] and differ among cell types [7–9] and species [9–12]. Therefore, it is especially interesting to examine RNA editing from an evolutionary perspective and as a possible mechanism of adaptation. With recent advances in high-throughput sequencing, the ability to sequence both the genomes and transcriptomes of organisms can be performed relatively cheaply and quickly, thus allowing us to delve into this area in more detail. With a focus on humans and other primates, flies, and cephalopods, this review provides an overview of the insights into the evolution of RNA editing that have been obtained so far and the evidence that supports a role for it in adaptation.

## ADAR protein evolution

**Fig 1:**
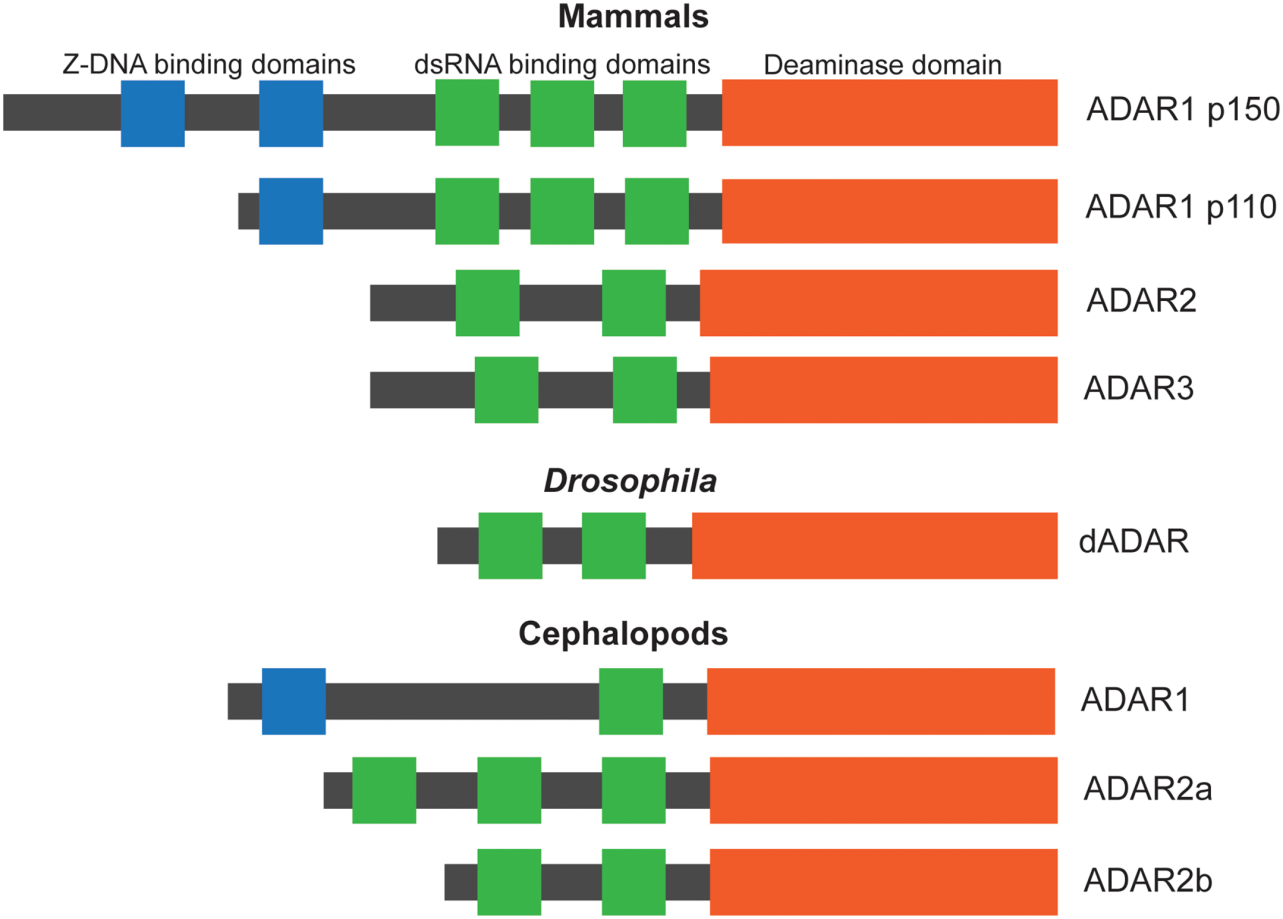
ADAR evolution in mammals, fruit flies, and cephalopods. Protein domains are represented by the large boxes and colored as follows: orange, deaminase domain; green, dsRNA binding domain; blue, Z-DNA binding domain. The domain structure shown for ADAR1 is that of octopus ADAR1 and that for ADAR2a is for squid. ADAR, adenosine deaminase acting on RNA; dsRNA, double-stranded RNA.

ADAR proteins exist in all metazoans. A common feature among all ADAR proteins is at least 1 N-terminal dsRNA binding domain and a C-terminal deaminase domain. Fig 1 shows domain structure diagrams of the ADAR proteins that exist in mammals, fruit flies, and cephalopods. Mammals have 3 main ADAR proteins. ADAR1 exists as 2 isoforms, p110 and p150 [13]. ADAR1 p110 has 2 Z-DNA binding domains, 3 dsRNA binding domains, and a deaminase domain [14–16]. ADAR1 p150, induced by interferons [17], has similar structural organization to the p110 isoform but contains an additional Z-DNA binding domain. ADAR2 and ADAR3 both contain 2 dsRNA binding domains and a deaminase domain [18–20]. Unlike ADAR1 and ADAR2, ADAR3 is not known to be catalytically active [20]. Cephalopods have both ADAR1 and ADAR2. Unlike human ADAR1, squid ADAR1 has only 1 dsRNA binding domain [21,22], while ADAR2 has 2–3 dsRNA binding domains, depending on the splice isoform. The extra dsRNA binding domain in squid ADAR2a increases RNA binding and editing activity [23,24]. *Drosophila* only has 1 ADAR, with 2 dsRNA binding domains and 1 deaminase domain [25]. Its protein sequence shares higher similarity to mammalian ADAR2 compared with ADAR1 or ADAR3 [25]. A study demonstrated that insects have completely lost ADAR1 and that human ADAR2 can edit *Drosophila* editing substrates as well as rescue many of the phenotypes of *Drosophila* ADAR mutants [26]. Taken together, these data suggest that *Drosophila* ADAR most closely represent mammalian ADAR2.

Although most studies examining RNA editing evolution and adaptation have focused on editing sites, it is worth discussing evolutionary analyses of the ADAR proteins themselves. A study examining human and primate ADAR sequences found evidence of purifying selection for all 3 ADAR proteins in both humans and primates, with ADAR1 being less evolutionarily constrained than ADAR2 and ADAR3 [27]. However, certain regions within the proteins demonstrated signatures of positive selection. For instance, in primates, positions in the N-terminal portion of the interferon-induced ADAR1 p150 isoform showed evidence of positive selection [27]. In humans, a position in the deaminase domain of ADAR1 that is next to mutations associated with Aicardi-Goutieres syndrome, as well as a homologous position in the second dsRNA binding domain of ADAR2 and ADAR3, also showed signs of selection [27]. Additional studies in modern human populations also showed that certain noncoding regulatory variants near all 3 ADAR genes are selected for [27]. Considering these human and primate results, as well as the fact that ADARs are present in all metazoan lineages, it is clear that ADARs play an important biological role in a diverse array of organisms.

## Comparing editing characteristics between species

**Fig 2:**
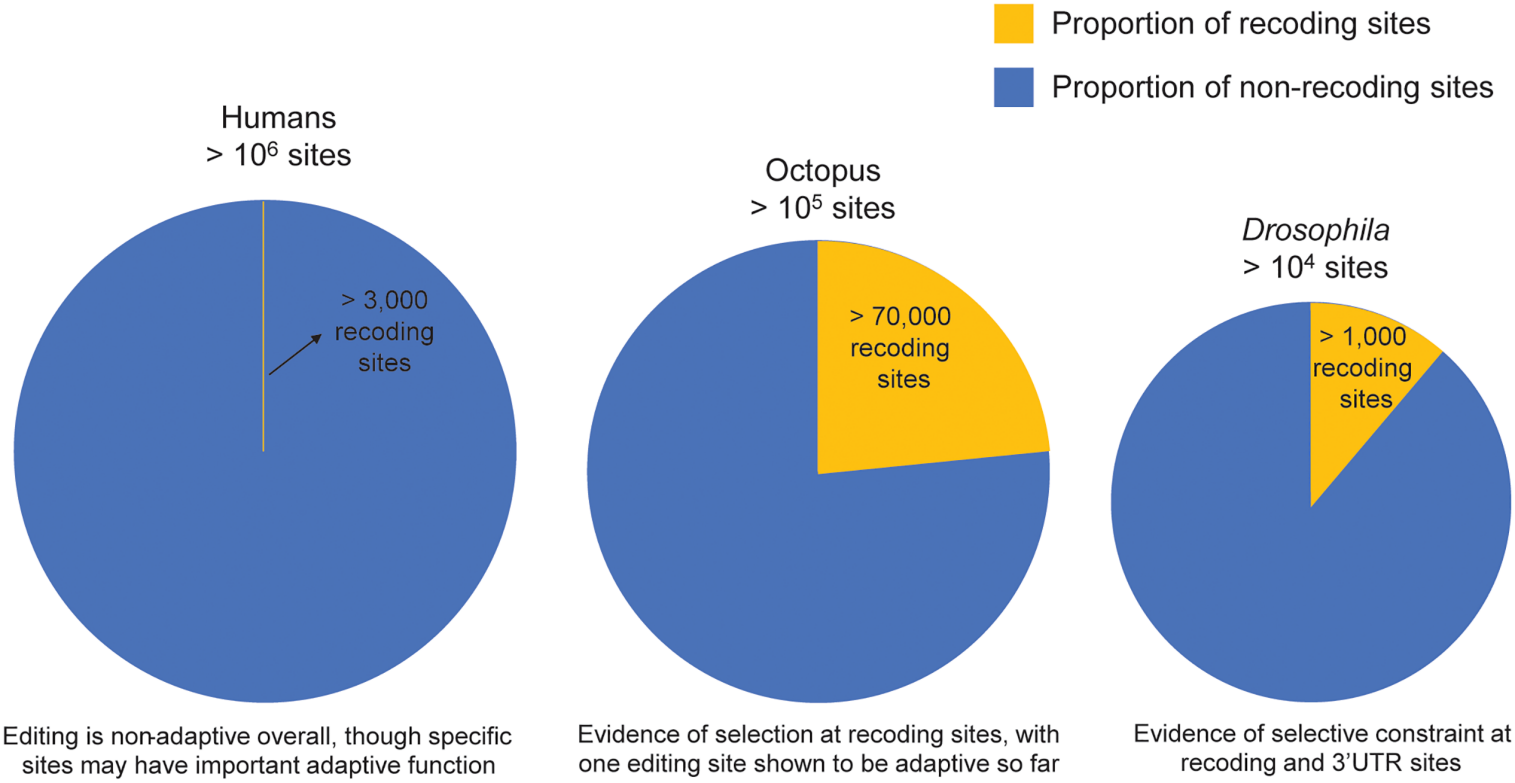
Comparing editing sites across species. Diagram showing the number and proportion of recoding editing sites in humans [30], octopus [31], and *Drosophila* [30,45]. For octopus, only sites in annotated regions were considered.

Given that A-to-I RNA editing exists in all metazoans, it is interesting to compare how different editing systems work across species. From the regulation to function of RNA editing, there are similarities in the patterns, dynamics, and phenotypes of editing in different species, suggesting a common evolutionary importance for it. For instance, in mice and flies, editing levels tend to increase throughout development, independently of ADAR gene expression [28,29]. This suggests the possibility of other *trans*-regulators of RNA editing, which may be shared among these different organisms. In regard to the specific sites that are edited and how they may affect protein sequences, the proportions of sites that are recoding (nonsynonymous) and non-recoding (synonymous or in noncoding regions) can vary from species to species (Fig 2). The vast majority of editing sites in humans and primates, for example, are in the ubiquitous inverted repeat *Alu* elements, which form stable dsRNA structures and are largely in noncoding regions of the genome [30]. However, one key similarity across many species is the existence of nonsynonymous sites in brain-related genes. In *Drosophila* and cephalopod species, these types of sites are extremely prevalent, making up a large fraction of their total editing sites [22,29,31]. Due to the prevalence of *Alu* editing sites, nonsynonymous sites are much less frequent in humans, making up <1% of total editing sites [30]. However, there are some key nonsynonymous sites that affect signaling in the brain, such as the glutamine to arginine (Q/R) site in glutamate receptor 2 (known as GluA2, GluR2, and Gria2) [32] and a glutamate to glycine site in the calcium-dependent activator protein for secretion 1 (CAPS1) [33]. Also, a conserved voltage-gated potassium ion channel motif has been found to be edited at various sites in humans and *Drosophila* [34,35]. Along similar lines, organisms lacking functional ADAR protein often have neurological phenotypes. For example, worms have impaired chemotaxis [36]; flies experience locomotion defects, age-dependent neurodegeneration, and other neurological phenotypes [25]; and mice without ADAR2 experience seizures and die shortly after birth [37]. In humans, several neurological diseases, including ALS [38,39], autism [40], depression [41], epilepsy [42,43], and schizophrenia [44], have also shown to be associated with altered editing levels. Thus, while the same sites are not always edited in different species, editing of recoding sites in all of these species is strongly associated with neurological function.

## Human and primate RNA editing evolution

With millions of editing sites in primates and humans, largely due to the expansion of repetitive *Alu* sequence elements in the primate lineage, one important question to ask is whether there is any evolutionary advantage to having all—or any—of this editing. Many efforts have been undertaken to determine signatures of selection near editing sites and identify which ones are likely to be adaptive. For instance, one study found purifying selection around editing sites in humans and rhesus macaques, with more constraint around coding sequences [46]. Editing in noncoding regions may be important as well; another study also on human and rhesus macaques found that piwi-interacting RNAs (piRNAs) containing editing sites in *Alu* elements were more evolutionarily constrained than more distal regions in the same *Alu* element, suggesting a crosstalk between the 2 pathways [47].

Although there appears to be high conservation around certain editing sites in humans and primates, there still remains the question of whether these sites are adaptive. In general, one expects that editing sites that recode for nonsynonymous amino acid changes, are edited at high levels, and are in evolutionarily constrained regions of the genome are more likely to be advantageous. However, it appears that, in general, RNA editing in humans is deleterious and thus nonadaptive. One study provided several lines of evidence to demonstrate this. The authors found that nonsynonymous editing sites are edited at lower levels and are fewer in number compared with synonymous sites, editing is rarer in essential genes, and genes under purifying selection or with high expression have lower editing levels [48]. In addition, as stated earlier, one important aspect of RNA editing that differentiates it from amino acid changes is that it contributes to the diversity of the transcriptome. As such, one expects that if RNA editing has an overall advantage, edited adenosines (As) in general should not frequently be replaced with guanosines (Gs) throughout the evolution of different species. However, this study also found that edited As are more likely to be mutated to Gs than unedited As. One caveat to this argument is that the presence of G mutations at editing sites suggests that the organism can indeed tolerate either A or G nucleotides at this position, so the site may not be deleterious. In addition, the G at the editing site may even be more beneficial than the A and could act as a precursor to a genetically encoded G at that position. The study also showed that editing sites that were replaced with Gs or other nucleotides to generate several different types of amino acids were shown to have higher editing levels than all other categories of editing, including conserved sites with no interspecies SNPs, supporting the claim that editing may have a deleterious effect on protein function [48]. Other separate studies examining human RNA editing made several similar observations, including that editing sites in conserved regions show significantly lower editing levels [49] and that both synonymous and nonsynonymous sites are in regions with lower conservation than average [27].

Although human RNA editing was not shown to have an adaptive role overall, it is still possible that specific individual editing sites have a positive functional effect. Recent efforts have utilized evolutionary comparisons to determine which sites in humans are likely to be functionally important. One study identified human sites that had high homology to mouse and found 59 highly conserved sites that were also present in rat, cow, opossum, and platypus, 34 of which were nonsynonymous [50]. They observed that these sites had higher editing levels compared with nonconserved sites and were over-represented in genes with nervous system function [50]. Another study built off this analysis and examined nonsynonymous editing sites that are conserved, hardwired, unfound, or diversified in humans and 44 other vertebrate species, which included the 34 nonsynonymous sites from the previous study. They found that a significantly higher fraction of conserved and hardwired sites contained human–mouse shared editing sites, compared with the unfound or diversified sites [51]. In addition, the shared editing sites in the conserved and hardwired groups had significantly higher editing levels than the unshared sites, though this was not the case for the unfound and diversified sites [51]. Therefore, this study proposes that these conserved and hardwired nonsynonymous sites that have high editing levels are likely to be beneficial and can serve as candidates for future functional validation studies. Some additional efforts to identify functionally important editing sites will be described in the “Adaptive Function of RNA Editing Events” section.

## *Drosophila* RNA editing evolution

*Drosophila* is a convenient and useful model organism to study the role of RNA editing in evolution and adaptation. Because many editing sites in *Drosophila* code for nonsynonymous changes in brain-related proteins, contrasting it greatly with the editing landscape in humans, this raises the possibility that RNA editing is more likely to play an adaptive role in *Drosophila* flies. Examining the extent of conservation in these editing sites and surrounding regions can provide insights into their evolutionary importance. One study analyzed several nonsynonymous editing sites in *Syt1*, which encodes a calcium-binding synaptic vesicle protein involved with neurotransmitter release. By examining regions of conservation in *Syt1* in 34 insect species, this study found that RNA structural changes across more than 250 million years of evolution modulate *Syt1* editing levels by affecting the underlying RNA structure [52]. These sequence elements, also known as editing complementary sequences (ECSs), can maintain high editing levels by forming dsRNA structures with the region surrounding the editing site(s) of interest. Since this study was published, the ECSs for several hundred more editing sites have been predicted by identifying regions of sequence conservation that are predicted to form dsRNA structures with conserved editing sites [45].

Editing sites in conserved regions are candidates for RNA editing adaptation, but further characterization of them is still needed to clarify their role in it. For instance, it would be important to know whether they are enriched with highly edited, nonsynonymous sites and whether they show significant enrichment of any gene ontology or functional categories. Three recent papers studying the evolution and adaptation of RNA editing in *Drosophila* shed some light on this [6,11,53]. Although the papers used different methods, a general theme for all is that certain sites (specifically nonsynonymous, conserved sites) are likely to be functionally important and adaptive and are enriched in genes involved in nervous system function. Two of the studies claimed evidence of positive selection for certain editing sites by comparing nonsynonymous and synonymous editing; one demonstrated that editing events in conserved genes are enriched for nonsynonymous sites compared with synonymous sites [53], while the other showed that the frequency of nonsynonymous editing is significantly higher than that of synonymous editing and also higher than expected under neutral conditions [6]. The third study showed that there is higher editing and more clustering in evolutionarily older sites and that both nonsynonymous and 3’UTR sites show higher constraint and higher editing levels [11]. In ADAR mutants, genes with edited 3’UTRs also showed increased expression compared with genes edited elsewhere [11]. Together, these results suggest that RNA editing plays a larger role in adaptation in *Drosophila* than in humans, although more work needs to be done to clarify the functional importance of this pool of conserved and highly edited sites.

Another interesting thread of research is the examination of how RNA editing is used in adaptation to different environments. Several studies of *Drosophila* have demonstrated that temperature affects editing levels [5,6,54], though the importance of this phenomenon is not entirely clear. One study recently shed light on this area; after entraining wild-type and ADAR hypomorph mutant flies to 18°C or 29°C, it found that ADAR hypomorph mutants have less drastic gene expression changes between 18°C and 29°C compared with wild-type flies, suggesting that having lower ADAR activity and editing levels leads to a weaker transcriptional response and adaptation to a change in temperature [54]. The importance of editing for temperature adaptation extends to the behavioral level as well; though wild-type and ADAR hypomorph flies displayed similar locomotor activity at 18°C in both light and dark conditions, the mutants showed significantly lower locomotor activity in dark conditions at 29°C compared with the wild-type flies at the same temperature [54]. Together, these results suggest that editing alterations in response to temperature are not merely a consequence of the shift in temperature but may be needed to properly adapt to the change in temperature. The mechanisms underlying temperature adaptation of RNA editing will be discussed further in the “Regulation of Adaptive RNA Editing” section.

## Cephalopod RNA editing evolution

Perhaps the most intriguing and fruitful studies of RNA editing evolution and adaptation have come from cephalopods, such as squid and octopus. RNA editing in cephalopods is extensive; for example, squid have over 57,000 editing sites, affecting most transcripts in the nervous system [22], and many thousands of editing sites have also been identified in the octopus [21,31]. In addition, the only verified example of RNA editing adaptation that has been characterized to date is a nonsynonymous editing site in potassium voltage-gated channel subfamily A member 1 (Kv1.1) in the octopus [55]. Octopuses that live in cooler water temperatures had higher editing levels at this site, while those in warmer water temperatures had lower editing levels. Biophysical assays in which the edited and unedited versions of the potassium channel were expressed in *Xenopus* oocytes showed that high editing levels destabilized the channel’s open state, thus accelerating the closing rate of the channel [55]. Therefore, it appears that high editing in this channel evolved in the octopus in cooler waters to speed up its gating kinetics, a way of adapting to the lower water temperature.

Given this exciting result, it is natural to wonder how many of the thousands of other editing sites in cephalopods are involved in adaptation. A recent study has shed some light on the conservation of editing sites across the genomes of these organisms and characterized this editing in more detail. By analyzing editing levels in cephalopods (octopus, squid, and sepia), as well as molluscs (nautilus and sea hare), the authors found extensive conservation of nonsynonymous editing sites across the different cephalopod species [31]. In addition, the fraction of sites that are nonsynonymous is higher than expected and increases as editing levels increase, providing evidence of adaptation. Similar to *Drosophila*, cephalopods have increased conservation (both between species and within species) in the regions around conserved editing sites, which is likely needed to form dsRNA structures that are important for editing to occur. The function of a few specific sites in potassium Kv2 channels was also investigated by injecting edited and unedited isoforms in *Xenopus* oocytes. Interestingly, for a site common to all 3 cephalopod species studied, the unedited form of the channel had different closing rates between the species, while the edited form showed similar closing rates for all the species [31]. Considering the vast amount of editing in cephalopods and these exciting initial results of the adaptation and evolution of editing in these organisms, there are likely to be many more intriguing results to bring us closer to knowing the extent of RNA editing’s role in these processes.

## Regulation of adaptive RNA editing

**Fig 3:**
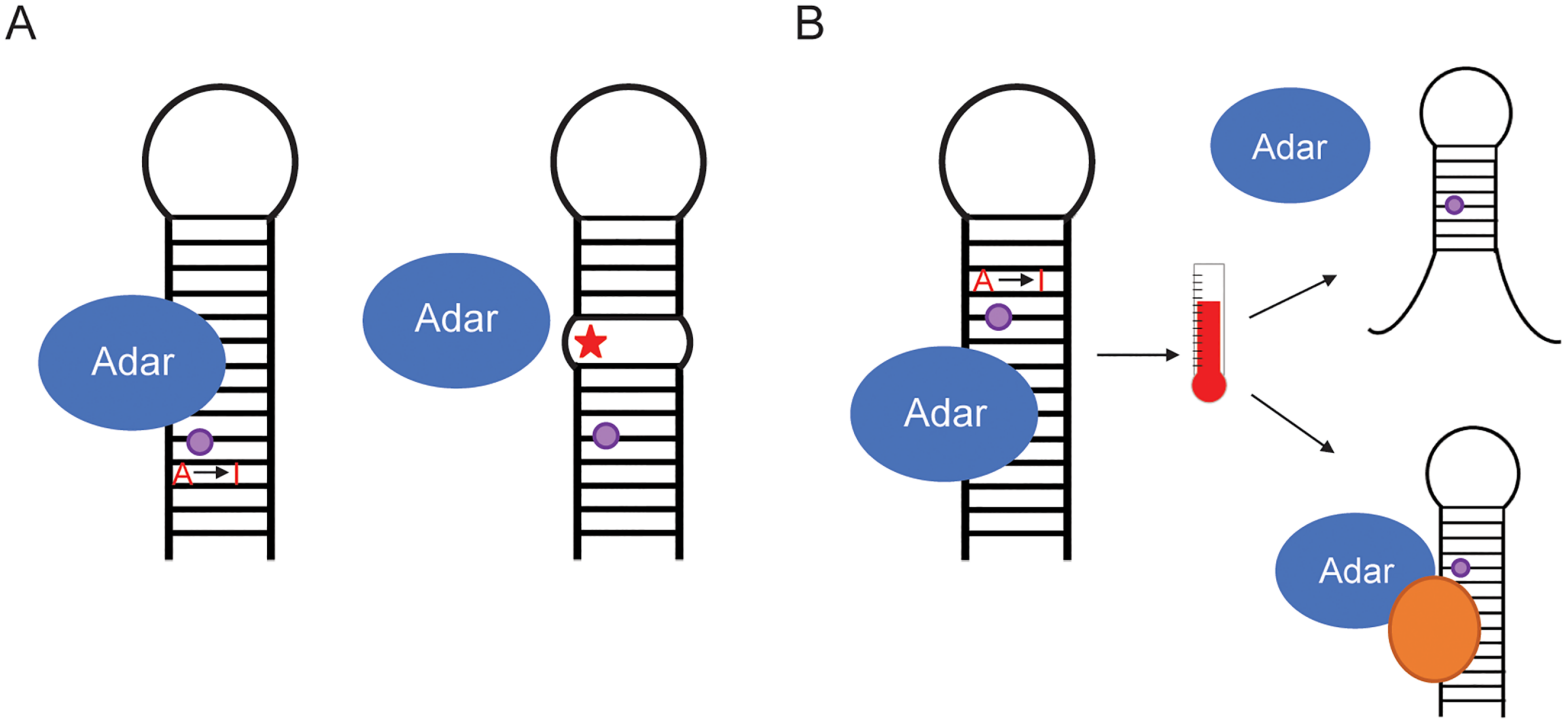
Comparing genetic and environmental regulation of editing. (A) In genetic regulation, a mutation (represented by the red star) may be near an editing site (purple dot) and alter the underlying RNA structure. This could affect ADAR’s ability to bind and edit the RNA. (B) In environmental regulation, or more specifically temperature regulation, an increase in temperature may destabilize the RNA structure containing the editing site or affect the expression of other *trans*-regulators of RNA editing. ADAR, adenosine deaminase acting on RNA.

We know that RNA editing can be adaptive, as demonstrated in the Kv1.1 potassium channel in the octopus. However, it is unknown whether the increase in editing in cold-adapted octopuses is caused by genetics or environment. If it is a genetically encoded adaptation, the cold-adapted octopuses may have 1 or more genetic mutations that increase the RNA structure stability of the editing substrate, thus allowing editing to occur more efficiently (Fig 3A). If it is a more transient acclimation to environment, the lower temperature itself may lead to increased RNA structure stability at this site or perhaps cause altered expression of *trans*-regulatory factors of RNA editing (Fig 3B). Both factors could be involved simultaneously as well.

Recent studies in *Drosophila* have shed light on the environmental and genetic contributions to regulating editing levels, which help inform the role they may play in adaptation. As alluded to previously, a few studies have demonstrated that temperature affects editing levels at dozens of sites [5,6,54], and this temperature regulation of editing appears to be evolutionarily conserved [5,6]. Most sites have decreased editing at higher temperatures, which is likely due to RNA structure destabilization, making ADAR’s binding and editing less efficient [5,6,54]. Additionally, sites edited at 18°C and 25°C were observed to be in regions of higher evolutionary conservation and were more likely to be in predicted dsRNA structures than sites edited at 29°C [54], suggesting that editing evolved to be more efficient at lower temperatures. ADAR protein levels are also decreased at 30°C compared with 20°C, though not at 20°C compared with 10°C, which suggests another possible reason for the editing level decrease [5].

There is also evidence that genetics contributes to editing level differences, not only between flies within a common environment but also between fly species and flies living in different microclimates. In particular, one study experimentally verified that an intronic SNP in the gene *prominin* contributes to its editing level differences in flies from different microclimates, though it is unknown if the change in editing is adaptive [56]. As with temperature, genetic mutations may affect editing levels by altering the RNA structure of the editing substrate. Both genetics and temperature may also interact to regulate editing levels. Although these results provide valuable insights into possible mechanisms behind RNA editing adaptation, further work is needed to validate them.

## Adaptive function of RNA editing events

**Fig 4:**
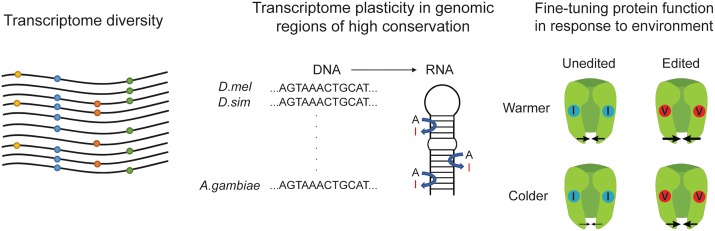
Advantages of RNA editing for adaptation. RNA editing may be advantageous for adaptation because it contributes to transcriptome diversity, generates plasticity in genomic regions of high conservation, and can be used to fine-tune protein function in response to the environment. *A*. *gambiae*, *Anopheles gambiae*; *D*. *mel*, *Drosophila melanogaster*; *D*. *sim*, *Drosophila simulans*.

One intriguing question is how RNA editing differs from SNPs: what is the advantage of producing changes with RNA editing through tweaks in RNA sequence and secondary structure, sometimes thousands of bases away, rather than directly modifying a single base at the DNA level? One possible advantage is that RNA editing can generate more diversity than DNA mutations within transcript sequences by producing a plethora of editing isoforms (Fig 4). In addition, as mentioned previously, the regions around beneficial editing sites may be conserved to maintain the dsRNA structure needed for editing to occur. However, another way to view this is that RNA editing can aid adaptation by allowing for modifications of highly important, conserved parts of the genome (Fig 4). Indeed, individual RNA editing events in a few highly conserved genes have been found to be functionally important. The most striking example of these is the Q/R site of the GluA2 glutamate receptor. The region around this site is conserved between human and mouse, and without RNA editing at this key site, mice experience a calcium ion influx, motor neuron death, seizures, and an early death [37]. Studies in cephalopods and *Drosophila* have also suggested that there is a tradeoff between genome evolution and transcriptome plasticity; in other words, advantageous editing is likely to occur in highly conserved, slowly evolving regions of the genome [6,11,31,53]. Though one adaptive editing site has already been demonstrated in the octopus [55], it would be interesting to examine additional RNA editing events in highly conserved regions that may be providing an adaptive advantage.

The octopus Kv1.1 RNA editing event highlights another important advantage of RNA editing: unlike hard-coded SNPs, RNA editing can affect only a fraction of transcripts and thus facilitate fine-tuning (Fig 4). One example of the tuning of editing levels is “autoediting” of ADAR, in which the ADAR protein edits ADAR transcripts to produce a different splice form (mammals) [3] or an isoform with a different amino acid (*Drosophila*) [25], thus altering the resulting protein activity. Autoediting is functionally important; in *Drosophila*, the overexpression of the unedited form of ADAR is lethal [57]. Editing may also assist in the fine-tuning of the biophysical properties of ion channels; though only the editing of the octopus Kv1.1 channel is known to be adaptive in this manner [55], editing can alter the properties of several ion channels in *Drosophila* as well [58–60]. As the function of additional editing sites is studied, there are likely to be more examples of how RNA editing plays a role in fine-tuning protein function.

As stated previously, the majority of RNA editing events in humans and primates do not appear to be precisely targeted to recoding sites like in the GluA2 receptor transcript but rather occur in large batches in regions of *Alu* sequences [61]. Interestingly, humans and primates are not the only organisms with vast amounts of editing in noncoding regions; the coral *Acropora millepora*, a basal metazoan with one of the most primitive nervous systems, was recently discovered to have over 500,000 A-to-I editing sites, with the majority located in noncoding regions [62]. This suggests that editing may have initially arisen as a general mechanism to mutate and destabilize dsRNA structures rather than to generate specific mutations in coding regions [62]. Despite this, the functional importance of vast amounts of editing in noncoding regions is not entirely clear. One idea is that RNA editing in *Alu* elements, which have high sequence similarity, may be functionally important in increasing transcriptome diversity (Fig 4). More conserved *Alu*s tend to be in neuronal genes [63], so it is possible that *Alu*s are neurologically functional and RNA editing plays a critical role in generating the high level of variation needed to produce a complex neurological system. Another functional role of editing may be the repression of harmful innate immune responses activated by *Alu* editing. The removal of RNA editing by ADAR1 leads to a harmful interferon induction caused by dsRNA sensor activation [64]. Because inverted *Alu*s can form long dsRNAs, there may be a need to suppress this interferon induction when *Alus* are expressed. Editing *Alu* sequences could have additional advantageous functions in primate evolution, for instance by creating new splice sites for exonization, regulating editing in nearby coding sequences, and modifying miRNA binding and gene expression if they are located in 3’UTRs [65]. Another important point is that editing of *Alu* RNAs may keep them in the nucleus, which could prevent them from interfering with protein translation [66,67]. Further work is needed to determine how many *Alu* editing sites, out of millions, are functionally important and involved in adaptation.

## Conclusions

With the identification of thousands of new editing sites in many different species, we can begin to determine the functional importance of editing, whether editing plays a role in adaptation, and how editing might differ between these species. The overall neurological role of editing appears conserved because studies in a variety of species have found that at least some neuronal nonsynonymous editing events are likely important. However, the extent of this varies vastly across different species. In humans, a few nonsynonymous editing sites do affect protein function. However, the fact that most editing is in noncoding *Alu* regions and seems to be nonadaptive overall suggests that most editing is not functionally important, although the function of noncoding and *Alu* RNA editing is an open area of research. Editing seems to play more of an adaptive role in *Drosophila* and cephalopods, which have many more nonsynonymous editing sites compared with humans; additionally, many of these sites show evolutionary constraint. Although some of these sites—mainly nonsynonymous sites in ion channels—have been shown to affect the biophysical properties of these proteins, it is unclear whether these editing levels change as an adaptive response. In cephalopods, we do have an example of a temperature-adaptive editing site [55]. With extensive recoding editing sites in many neuronal transcripts, it is likely that there are many more examples of this, especially because these sites show strong signatures of selection.

The results discussed in this review provide valuable insights into the evolution and adaptation of RNA editing, but some details still need to be filled in. For example, which sites not only alter their levels in response to environmental conditions but serve a functional purpose in doing so? In other words, how many other sites are similar to the adaptive site in the octopus Kv1.1 channel, both in the octopus and other animals? Though efforts to answer this question have been described, it is perhaps most beneficial to examine how editing levels differ in organisms from diverse environments. It may be especially helpful to examine other poikilothermic species because their body temperatures are associated with the temperature of their surroundings. Environmental variables besides temperature may regulate editing as well. If the editing events that do change in response to the environment are in conserved or constrained regions—or are predicted to affect protein levels or function—then their functions could be examined in further experiments. Although there are various ways to test this, the development of the gene editing CRISPR/Cas9 system has provided a convenient way to mutate editing sites in vivo so that these sites are fully edited or unedited. If organisms with different editing levels at a particular site show some phenotypic difference, this suggests that the editing site is functionally important. Another area that would be interesting to investigate further is the regulatory mechanisms underlying adaptive RNA editing sites. As mentioned earlier, while genetics and environment are involved with modulating editing levels, likely through altering RNA structure, their role in RNA editing adaptation is not completely clear; it is possible that these mechanisms differ for various organisms and environments. One intriguing scenario in which both factors are involved is that when an organism is faced with a new environment, such as a change in temperature, a change in editing occurs as an initial acclimatization response. However, if that change in editing proves to be beneficial for the organism, a genetic mutation could potentially make that change in editing “permanent,” thus leading to adaptation. Measuring the editing levels of these adaptive sites at various temperatures and in organisms of different genetic backgrounds would provide greater insight into this area. With these results, the full extent to which RNA editing is involved in adaptation and the underlying mechanisms of this process can be determined.
